# Roles of Protein Disulfide Isomerase in Breast Cancer

**DOI:** 10.3390/cancers14030745

**Published:** 2022-01-31

**Authors:** Suhui Yang, Chanel Jackson, Eduard Karapetyan, Pranabananda Dutta, Dulcie Kermah, Yong Wu, Yanyuan Wu, John Schloss, Jaydutt V. Vadgama

**Affiliations:** 1 Division of Cancer Research and Training, Department of Medicine, Charles R. Drew University of Medicine and Science, Los Angeles, CA 90059, USA; eduardkarapetyan1@cdrewu.edu (E.K.); pranabandutta@cdrewu.edu (P.D.); yongwu@cdrewu.edu (Y.W.); yanyuanwu@cdrewu.edu (Y.W.); johnschloss@cdrewu.edu (J.S.); 2 School of Pharmacy, American University of Health Sciences, Signal Hill, CA 90755, USA; 3 Post Baccalaureate Pre-Medical Program, Charles R. Drew University of Medicine and Science, Los Angeles, CA 90059, USA; chaneljackson@cdrewu.edu; 4 Urban Health Institute, Charles R. Drew University of Medicine and Science, Los Angeles, CA 90059, USA; dulciekermah@cdrewu.edu; 5 Jonsson Comprehensive Cancer Center, David Geffen School of Medicine, The University of California at Los Angeles, Los Angeles, CA 90059, USA

**Keywords:** breast cancer, triple-negative breast cancer, protein disulfide isomerase, protein disulfide isomerase inhibitor

## Abstract

**Simple Summary:**

Triple-negative breast cancer (TNBC) is the most aggressive subtype of breast cancer and has a poor prognosis and higher recurrence rate due to ineffective therapy. Even with newly approved therapeutics, only limited TNBC patients could have benefited from the regimens. Protein disulfide isomerase (PDI) has been of great interest as a potential therapeutic target for cancers due to its impacts on tumor progression, metastasis, and clinical outcomes. Here, we discuss the roles of PDI members in breast cancers such as TNBC and the PDI inhibitors studied in breast cancer research.

**Abstract:**

Protein disulfide isomerase (PDI) is the endoplasmic reticulum (ER)’s most abundant and essential enzyme and serves as the primary catalyst for protein folding. Due to its apparent role in supporting the rapid proliferation of cancer cells, the selective blockade of PDI results in apoptosis through sustained activation of UPR pathways. The functions of PDI, especially in cancers, have been extensively studied over a decade, and recent research has explored the use of PDI inhibitors in the treatment of cancers but with focus areas of other cancers, such as brain or ovarian cancer. In this review, we discuss the roles of PDI members in breast cancer and PDI inhibitors used in breast cancer research. Additionally, a few PDI members may be suggested as potential molecular targets for highly metastatic breast cancers, such as TNBC, that require more attention in future research.

## 1. Introduction

Breast cancer is the most common malignant tumor and the second leading cause of cancer-related death in women. In the United States, it is estimated that about 30% of newly diagnosed cancers in women will be breast cancer, and about one in eight women (13%) will develop invasive breast cancer over their lifetime [1]. Breast cancer is genetically and epigenetically not just one disease, but a diverse group of disorders with various clinical features [2]. Most breast cancer (about 81%) is invasive cancer. It can be further classified into four subtypes: Luminal A, Luminal B, HER2-enriched, and Basal-like, depending on the presence of hormone receptors and human epidermal growth factor receptor 2 (HER2) [3]. Basal-like breast cancer is commonly known as triple-negative breast cancer (TNBC). The term “triple-negative” in TNBC comes from its unique composition of lacking three receptors; the majority of TNBC patients lack expression of the estrogen receptor (ER) and the progesterone receptor (PR) and overexpression or amplification of HER2 [4]. TNBC is a more aggressive type of breast tumor because it grows rapidly and is more likely to spread, resulting in high metastatic potential [5,6]. TNBC also has higher recurrence rates even after being treated with chemotherapy, which is the mainstay for TNBC treatment [6,7,8,9]. These features make TNBC represent over 50% of mortality in breast cancer, whereas it accounts for 15–20% of all cases [10]. In addition, health disparities have been highlighted in breast cancers, most probably due to TNBC. In detail, the aggressive TNBC subtype of breast cancer is identified more frequently in African-American women in advanced stages than Caucasian-American women [11,12,13], and African-American women exhibit the lowest survival rate of any race or ethnic group in the same cancer stage [13].

## 2. Recent Therapeutic Options and Molecular Targets in TNBC

**Figure 1:**
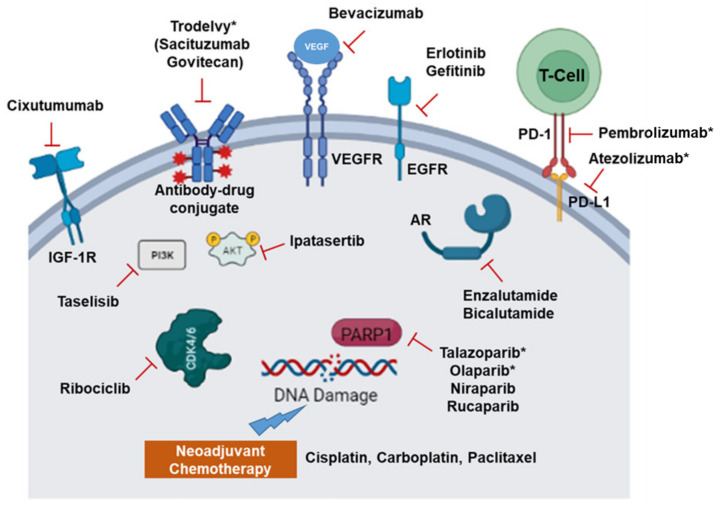
Molecular targets in TNBC. Inhibitors of pathways discussed in the text are shown with inhibitors or antibodies. These include inhibitors targeting EGFR, VEGFR, and AR. Checkpoint inhibitors such as anti-PD-1 and PD-L1 are used to inhibit cancer cell and T cell interactions. Neoadjuvant chemotherapy and PAPR inhibitors are used to abrogate DNA Damage Response. (*) indicates U.S. Food and Drug Administration (FDA) approved drugs for treating TNBC.

TNBC is an aggressive disease with fewer specific targets due to the lack of any receptor expression. TNBCs tend to occur in younger women and have a higher potential to metastasize to distant organs or for regional relapse. In a study involving 2534 breast cancer patients, 35% of patients developed metastasis after 6 years: 15% to the brain and 14% to the lung [14]. Recently, research has expanded the targetable vulnerabilities in TNBC (Figure 1 and discussed below). Clinical efforts targeting multiple pathways, including DNA damage response, Epithelial-mesenchymal transition, (Wingless/Int-1) Wnt Signaling, Phosphatidylinositol-4,5-Bisphosphate 3-Kinase Catalytic Subunit Alpha (PIK3CA), and Androgen signaling, are currently ongoing, some of which are discussed below [15].

The standard of treatment for TNBC is neoadjuvant chemotherapy, which yields better pathologic complete response (pCR) [16]. Particularly, platinum-based chemotherapy with cisplatin or carboplatin alone or in combination has achieved pCR in a large number of patients [17]. More recently, targeted delivery with an antibody-drug conjugate has shown promising results. One such drug, Sacituzumab Govitecan, has been approved by the U.S. Food and Drug Administration (FDA) for the treatment of metastatic TNBC [18]. The drug combined cell surface human trophoblast cell-surface antigen 2 (Trop 2) antibody with a topoisomerase I inhibitor. However, TNBCs relapse at a higher rate compared to other types of breast cancer, as shown by lower Disease-Free Survival (DFS), which is associated with neoadjuvant chemotherapy and residual disease [19].

The early breakthrough treatment option is the use of Poly-ADP Ribose Polymerase (PARP) inhibitors after discovering its pathways in DNA repair. Incidentally, TNBCs have higher BRCA1 loss-of-function mutations or BRCAness, a clinical signature similar to sporadic BRCA1 or BRCA2 mutation [20,21]. Loss of BRCA or BRCAness is associated with a defective DNA damage response, since BRCA1 is involved in double-stranded DNA breaks. Thus, in the context of BRCA loss, PARP inhibition leads to the accumulation of single-strand breaks, ultimately causing cancer cell death. This is attributed to the synthetic lethality of PARP inhibition with BRCA1 loss-of-function mutations. With this rationale, the targeted groups of PARP inhibitors are the BRCA1 negative TNBC patients [22]. PARP inhibitors such as Olaparib, Niraparib, and Rucaparib are currently approved by the FDA for ovarian cancers, endometrial cancers, and castration-resistant prostate cancer. Currently, the only approved PARP inhibitor for TNBC is Talazoparib (Talzenna, Pfizer) [23]. It is approved as a single-agent therapy for the treatment of HER2-negative breast cancer with a germline BRCA1 mutation, and it shows significant improvement in progression-free survival compared to standard chemotherapy. Clinical trials of other PARP inhibitors, such as Olaparib, in combination with radiation or a monoclonal antibody Durvalumab for TNBC are underway (NCT03109080 and NCT03801369, respectively).

Besides the single target, the most recent efforts for TNBC treatment focus on the utilization of combination therapy. These include standard platinum-based therapeutics as a neoadjuvant or with mechanistically different chemotherapeutics. For example, two clinical trials have used this strategy when BRCA1/2 germline or somatic mutations are present. These trials have utilized Carboplatin with Docetaxel [24] or Doxorubicin with cyclophosphamide as opposed to neoadjuvant cisplatin (INFORM trial) [25]. Carboplatin has also been combined with an anti-microtubule agent such as Ixabepilone with a good prognosis in metastatic TNBC [26].

Other treatment efforts are on the way with different antibodies targeting mitogens such as Vascular Endothelial Growth Factor A (VEGF-A) (Bevacizumab) and small molecule inhibitors targeting kinases such as Epidermal Growth Factor Receptor (EGFR) (Erlotinib and Gefitinib) in TNBC [27,28]. More preclinical studies have shown that EGFR-targeted Chimeric Antigen Receptor (CAR) T cells could potentially be a treatment for TNBC, as these T-cells cause TNBC cell lysis [29]. TNBC has a high tumor mutation burden and thus is immunogenic. Along with that, the high levels of Program-cell Death-Ligand 1 (PD-L1) expression make TNBC a prime candidate for immune checkpoint inhibition therapy [30]. PD-L1 can cause T-Cell anergy (inactivation of T-cells as an immune tolerance mechanism), thus enabling TNBCs to evade immune detection. Therefore, checkpoint inhibitors such as Atezolizumab, an anti-PD-L1 monoclonal antibody, and a PD-1 receptor targeting humanized monoclonal antibody, such as Pembrolizumab, have been tested with neoadjuvant chemotherapy [31,32]. These combinations have shown significant improvement in overall survival. Additionally, PARP inhibitors such as Talazoparib have been tried in combination with Nab-Paclitaxel and anti-PD-L1 in BRCA mutated TNBC [33]. Additionally, the combination of PARP inhibitor Niraparib with Pembrolizumab, a Programmed death 1 (PD1) immune checkpoint inhibitor, has shown promising results in a preliminary trial with advanced and metastatic TNBC patients [34]. Other potential avenues are using inhibitors for Protein kinase B (AKT) (Ipatasertib), Aurora, and kinase inhibitor ENMD-2076, which have shown promising results [35,36]. In addition, Androgen Receptor (AR) antagonists Enzalutamide and Bicalutamide are in clinical trials in AR-positive TNBC patients. In the studies, the combinations of Enzalutamide with Paclitaxel (NCT02689427) or Bicalutamide with cyclin D1/CDK4 and CDK6 inhibitor Ribociclib (NCT03090165) are being used. AR inhibition has also been tried with a combination of PI3K inhibitor Taselisib, resulting in an overall increase in progression-free survival [37].

Multiple preclinical studies are exploring other avenues to target TNBCs to yield better outcomes. For example, the simultaneous targeting of EGFR, HER2, and HER3 with an antibody cocktail has shown tumor regression in patient-derived xenograft models and the downregulation of AKT and ERK pathways [38]. In vitro studies with Fingolimod, an inhibitor of sphingosine-1-phosphate, reduced TNBC cell growth in xenograft models [39]. Interestingly, intratumoral Toll-like receptor (TLR) 7/8 agonist 3M-052 has shown improvement in activating a tumor-immune microenvironment to protect against metastasis [40]. Other receptor tyrosine kinases, such as Insulin-like Growth Factor 1 Receptor (IGF-1R) targeting antibodies (Cixutumumab), are currently showing potential in the treatment of TNBC in pre-clinical studies with xenograft [41].

Together with the multiple preclinical studies, improved and promising outcomes from the currently approved therapeutics have also been reported. However, only limited TNBC patients could have benefited from the new therapeutic regimens. Thus, it still requires continuous effort to identify clinically relevant molecular targets for TNBC. Among the promising therapeutic targets for TNBC, PDI has emerged as an interesting molecular target for cancer research due to its critical role in the unfolded protein response (UPR) pathways. Thus, in this review, we provide an overview of PDIs in ER stress and UPR pathways, the roles of PDI family members in breast cancer, and the PDI inhibitors studied in breast cancer research.

## 3. Protein Disulfide Isomerase (PDI) Family

Disulfide (s-s) bonds are important for maintaining natural structures of proteins for their normal biological functions. The disulfide bond formation occurs between cysteine residues through the oxidation of thiol groups and is then rearranged to achieve the correct conformation [42,43]. The most abundant and essential enzyme in the ER is PDI. PDI functions as a dithiol-disulfide oxidoreductase and molecular chaperone which participates in the oxidation (formation), reduction (breakage), and rearrangement (isomerization) of disulfide bonds. PDI also assists in protein folding by preventing the aggregation of misfolded proteins [44].

**Figure 2:**
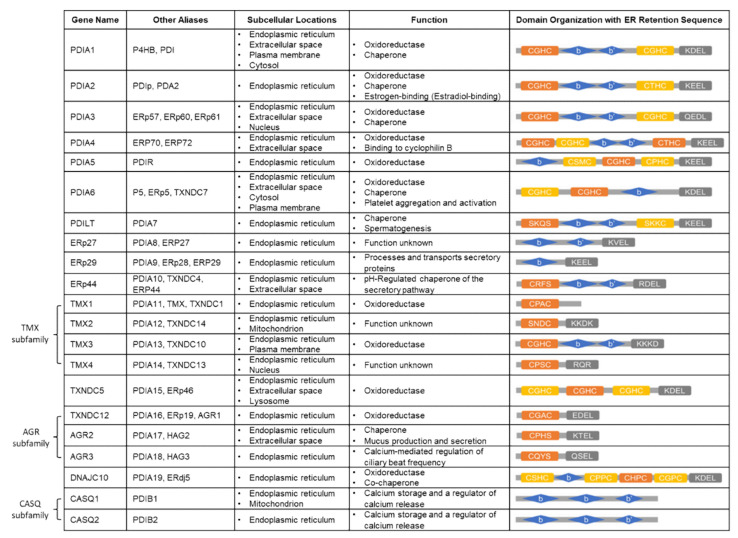
The human PDI gene family. Each gene is described with other aliases, subcellular locations, known functions, and domain organization. For the domain organization, the catalytically active a domains are represented in orange with active sites noted, active a’ domains are represented in yellow, b and b’ domains are represented in blue, and COOH-terminal ER retention sequences are represented in gray with their amino acid composition denoted. The figure was adapted and modified from Powell and Foster, Cancer Medicine 2021 [51].

The PDI family has at least 21 members that are resident in various cellular compartments, primarily within the ER and other cellular locations such as the nucleus, cytoplasm, or the plasma membrane. Although belonging to the same family, they show different lengths, different domain arrangements, and varied substrate specificity (Figure 2). The common domain that is present in all PDIs is the thioredoxin-like domain that can be further divided into two types (a and b) depending on the presence of a catalytic motif (Cys-X-X-Cys) [45,46]. The a-type catalytic domain, including a and a’, contains cysteines in the active sites that are thiol-reactive, and they are responsible for oxidoreductase activity [47]. The most conserved motif of CXXC is the CGHC (Cys-Gly-His-Cys) [48]. Catalytically inactive b-type domains (b and b’) that lack cysteines do not mediate disulfide bond formation. Instead, primarily, the b’ domain plays a role in recruiting substrates by constituting the principal substrate-binding site via hydrophobic interaction [49,50]. The archetype PDI protein, PDIA1, has a multidomain (a, b. b’, a’), a linker x, and the acidic C-terminal extension in which the ER retention signal resides. Other PDI members have a similar modular composition of thioredoxin-like domains in various arrangements, but there are some atypical members that only possess one type of domain. Depending on structural similarity, the PDI family can be divided into several subgroups, such as the PDIA, TMX, AGR, or CASQ subfamilies [45,51]. The typical PDIs (PDIA 1-6), PDILT, and DNAJC10 contain at least two active a-type domains (a and a’ domains) and two inactive b-type domains (b and b’ domains), except for PDIA5, PDIA6, and DNAJC10, which have one b domain instead of two b-type domains. The TMX (TMX1-4) and AGR (TXNDC12, AGR2-3) subfamilies carry only a-type domains, except TMX3, which contains b-type domains. TXNDC5 contains only a-type domains without the presence of a b-type domain. ERp27 and ERp29 have only b-type domains, whereas ERp44 contains a-type domains as well. The CASQ subfamily (CASQ1-2) possesses b-type domains and is the only PDI member without an ER retention sequence [45].

**Figure 3:**
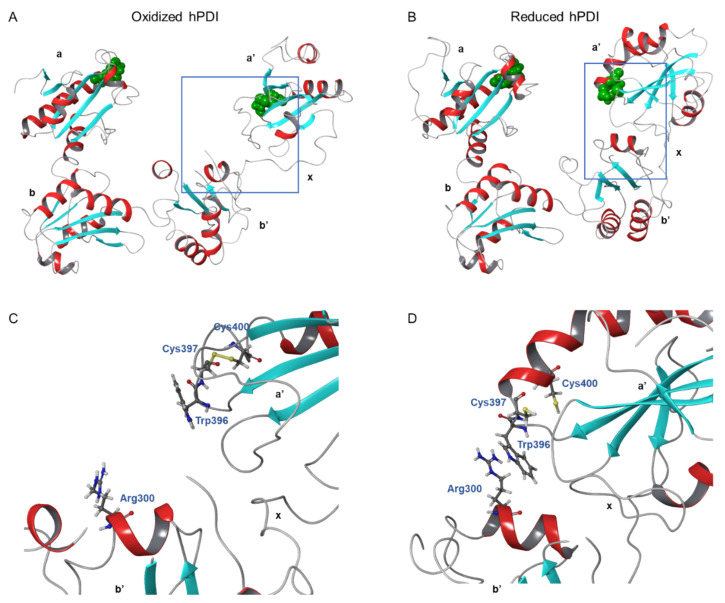
The overall structures of oxidized and reduced hPDI: (**A**,**B**) the crystal structure of oxidized human PDI (hPDI) (PDB #: 4EL1) (**A**) and reduced hPDI (PDB #: 4EKZ). Redox-active sites are represented as space-filling models in green; (**C**,**D**) the interaction between b’ and a’ domain of hPDI at different redox states. The Arg300 (b’ domain) and Trp396, Cys397, and Cys400 (a’ domain) are shown in ball and stick presentation. There is no observed interaction between Arg300 and Trp396 in the oxidized state (**C**), whereas the cation-π interaction between the guanidium of Arg300 and the indole ring of Trp396 is observed due to disulfide bond formation in Cys397 and Cys400 in the a’ domain (**D**). The a and a’ domains are catalytic domains, b and b’ domain are non-catalytic domains, and x is a linker. The figures were generated by using the Schrodinger program.

Each domain of the human PDI is arranged in a horseshoe shape with two CXXC active sites, with the a and a’ domains facing each other at the two ends [52]. In reduced PDI, a, b, and b’ domains are arranged on the same plane, and only the a’ domain twists outward by 45°. By contrast, all four domains are placed on the same plane in the oxidized state. The distance between the two redox-active sites in the a and a’ domains of the oxidized form increases to 40.3 Å compared to 27.6 Å of the reduced form, resulting in a more open hydrophobic inner space (14,400 Å^3^, two times larger than the area of reduced form, 6800 Å^3^) that is required for substrate binding (Figure 3) [52]. While almost no interaction is observed between the a and b domains, the redox-driven interdomain rotation occurs in the b’xa’ region [52]. An extensive cation-π interaction between the guanidium group of Arg300 in the b’ domain and the indole ring of Trp396 in a’ domain leads to a closed conformation in the reduced human PDI. However, in the oxidized state, oxidation of the CGHC motif of the a’ domain occurs. In other words, disulfide bond formation between cysteine residues (Cys397 and Cys400) in the a’ domain disrupts the cation-π interaction between Arg300 and Trp396 of the b’ and a’ domain, resulting in an open conformation. It allows PDI to capture unfolded and partially folded substrates, because oxidized PDI exists in an open state and assembles to form a face-to-face dimer. The resultant central hydrophobic cavity accommodates the substrate and efficiently introduces disulfide bonds into it [53]. Ultimately, correctly folded proteins with native disulfide bonds and hydrophilic surfaces are released from PDI. The oxidative folding in the oxidized state leads to higher chaperone activity than the reduced state [54]. The importance of the substrate binding on the proper oxidative folding of proteins has been demonstrated by using an inhibitor of PDI, bisphenol A (BPA), that binds to the substrate-binding site of PDI b’ domain. BPA causes significant spatial rearrangement and results in a more compact overall structure of PDI. This conformation switch leads to the subsequent closure of the substrate-binding pocket in the b’ domain, preventing PDI from binding to other proteins [55].

## 4. PDI in ER Stress and UPR Signaling

**Figure 4:**
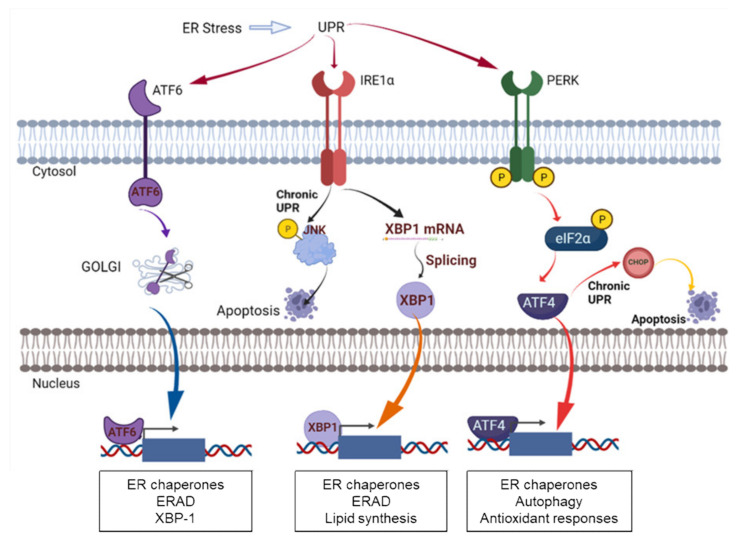
ER stress and the UPR pathway. The figure was adapted and modified from Powell and Foster, Cancer Medicine 2021 [51]. The figure was created with BioRender.com.

PDI works as a folding enzyme and a chaperone for disulfide bond formation, cleavage, and rearrangement in unfolded or misfolded proteins, so it is important for endoplasmic reticulum (ER) proteostasis. In other words, the dysregulation of PDI functions can disrupt protein folding efficiency in the ER lumen, leading to the accumulation of unfolded and misfolded proteins. This condition is known as ER stress, which further activates a cellular stress response called UPR (Figure 4). The UPR is carried out through ER stress sensors, inositol-requiring protein 1 (IRE1), activating transcription factor 6 (ATF6), and protein kinase RNA-like endoplasmic reticulum kinase (PERK) [56]. In the situation of ER stress, the process is initiated by the dissociation of glucose-regulated protein 78kDa (GRP78), known as binding immunoglobulin protein (BiP). Its dissociation allows ER stress receptors to be activated through the dimerization and phosphorylation of PERK and IRE1 or the cleavage of ATF6. Downstream signaling of these receptors induces chaperones that either facilitate protein folding to reduce the unfolded protein burden or remove the inappropriately folded proteins to further relieve ER stress [44]. In addition, PDI mediates regulation of the ER stress receptors upon activating the UPR pathway in response to ER stress [44,57,58]. For example, PDIA1 in its oxidized state is essential to activating PERK, whereas PDIA3 forms a complex with PDIA1 to let PDIA1 in its reduced state, which prevents PERK activation [59,60]. PDIA6 also prevents the activation of IRE1 by forming a covalent disulfide bond with the Cys148 of IRE1 [57,61]. On the other hand, PDIA5 regulates the ATF6 export from the ER and the activation of its target genes through disulfide bond rearrangement in ATF6 [62,63].

The early-stage UPR functions to alleviate ER stress and maintain cellular homeostasis. However, longer exposure to ER stress and prolonged UPR promotes cell death. Under chronic ER stress, PERK leads to the upregulation of proapoptotic C/EBP homologous protein (CHOP) expression [64]. Similarly, IRE1 also promotes the activation of the JNK pathway that interacts with the proapoptotic Bcl-2 members such as BAX and BAK [65].

Cancer cells are exposed to various stressors which increase the level of unfolded and misfolded proteins in the ER, triggering the activation of the UPR. The cancer cells, in turn, are in high demand for PDI in order to sustain rapid cell growth. The significance of PDI in supporting cancer cell survival is demonstrated by its upregulation in various cancers, including kidney, lung, brain, ovarian, melanoma, and prostate tumors. Interestingly, PDI overexpression is frequently correlated with tumor metastasis and invasiveness [66,67], chemoresistance [68,69], and lower survival rates in cancer patients [70].

## 5. The Functions/Roles of Specific PDIs in Breast Cancer

### 5.1. Overexpression of PDIs and the Role of PDI in Breast Cancer Proliferation

In breast cancer, PDI gene transcription is frequently upregulated. More specifically, out of 21 members, PDIA1, PDIA3, PDIA4, and PDIA6 are identified to exhibit an increased mRNA level in breast cancer, according to the Gene Expression Atlas dataset [51]. Protein analysis has revealed PDIA1 to be one of the most frequently upregulated proteins in breast tumor tissue, interstitial fluid, and, importantly, in the blood. The presence of high-level PDIA1 in the blood of breast cancer patients serves as a potential non-invasive serological marker for early detection [71]. Its high expression is observed in infiltrating ductal carcinomas of the breast in either sex [72,73] and in axillary lymph node metastatic breast tumors [74]. In breast cancer mammospheres, the protein levels of PDIA1, PDIA3 (ERp57), and ERp44 are elevated [75]. Another proteome analysis indicates that PDIA3 and PDIA6 show higher expression levels in invasive ductal carcinomas than in lobular carcinomas, and the high expression of PDIA3 and PDIA6 genes correlates with the aggressiveness of primary ductal breast cancer [76]. In addition, it is not surprising that AGR2 (PDIA17) is highly expressed in ER-positive breast cancer, as it mediates estrogenic actions [77]. It is evident in cell lines that AGR2 is present in MCF-7 (Luminal A) at a relatively high level but is low in MDA-MB-231 (TNBC) [67]. However, it is also involved in lobuloalveolar development due to its ability to stimulate cell proliferation [77]. In the recent proteomic study reported by Stojak et al., PDIA1 is identified as a major isoform of PDIs present in human breast cancer cells (MDA-MB-231 and MCF-7) [78]. PDIA3 (ERp57) is found to be the second most abundant isoform in both cell lines. PDIA4 (ERp72), PDIA6, and PDIA9 levels are high, but lower than PDIA1 and PDIA3. PDIs also show somewhat cell-type-specific expression. For example, PDIA1 and PDIA6 are highly expressed in breast cancer cells (MDA-MB-231, MDA-MB-468, and T47D), whereas PDIA4 (ERp72) expression is high in those two TNBC cells but moderate in T47D cells, indicating a complex transcriptional regulation mechanism [67]. Consistent with the findings, the mRNA levels of PDIA1, PDIA3, PDIA4, and PDIA6 are typically overexpressed in HER2-enriched and Basal-like breast cancer subtypes as evidenced by gene expression data attained from the Gene Expression Atlas datasets [79].

The overexpression of PDI is closely associated with breast cancer cell proliferation. Consequently, silencing PDI induces significant cytotoxicity in breast cancer cells such as MCF-7 [80]. PDI inhibitors such as PACMAs are known to interrupt the cell cycle progression in breast cancer cells [81]. PDI also affects the different mechanisms involved in breast carcinogenesis, including the estrogen receptor (ERα) or Wnt signaling pathways. PDI interacts with ERα, which mediates the proliferative effect of estrogens in breast cancer cells, but not with ERβ, which appears to be antiproliferative and negatively regulates the transactivation of ERα [82]. So, the knockdown of PDI in MCF-7 cells results in a significant increase in the ERβ/ERα ratio, possibly providing the beneficial effect of cancer prevention. Recently, PDI has been found to be vital for Wnt3a secretion and for the regulation of Wnt signaling, which is mainly involved in the processes of breast cancer proliferation and metastasis [83,84].

### 5.2. Role of PDI in Breast Cancer Invasion and Metastasis

Outside their roles in cell proliferation, PDI proteins are also involved in cancer cell adhesion and migration, affecting breast cancer metastasis. The transendothelial migration of cancer cells is initiated through the adhesion of the cells to endothelium. This process is regulated by cell adhesion receptors such as integrins. Integrins are composed of two subunits, α and β, and mediate the interaction between cell and extracellular matrix (ECM) proteins [85]. In patients with invasive breast cancer, the significant involvement of β_1_ and β_3_ integrins are observed. For instance, integrin α_2_β_1_ is upregulated in highly metastatic breast cancer cells like MDA-MB-231, compared to non-invasive breast cancer cells like MCF-7 [86]. A disintegrin and metalloproteinases (ADAMs) are a family of multifunctional proteins implicated in proteolysis and cell adhesion. The members, such as ADAM8, ADAM10, and ADAM17, are involved in breast cancer invasion and metastasis [87,88,89].

PDIs that are located at the cell surface interact with integrins and metalloproteases and regulate their functions. PDI facilitates the activation of integrins by catalyzing thiol-disulfide exchange on the cysteine-rich integrin extracellular domains. A similar mechanism by extracellular PDI is crucial for the activation of matrix metalloproteinases (MMPs) that are overexpressed in higher grades of breast cancer tumors and contribute to breast cancer metastasis [45,90]. On the other hand, when PDI interacts with ADAMs by catalyzing the thiol-disulfide exchange, it leads to a dramatic structural change. This change turns an open conformation of ADAMs in an active state into a closed conformation in an inactive state [91].

Multiple lines of evidence corroborate the involvement of specific PDIs in the process of breast cancer invasion and metastasis. Extracellular PDIA1 enhances the adhesion and migration of breast cancer cells (MCF-7 and MDA-MB-231) by potentially activating β integrins through thiol switches [78]. Also, PDIA1 increases cancer cell adhesion to the endothelial monolayer and collagen type I rather than PDIA3 [78]. The function of AGR2 to promote metastasis is demonstrated in a rat model of overexpressing AGR2 by enhancing the adhesive property of the cells [92]. Similarly, the extracellular AGR3 is found to increase the migratory properties of ER-positive breast cancer cells MCF-7 and T-47D [93]. Its regulation of breast cancer cell migration and adhesion is executed by inducing the phosphorylation of tyrosine kinases such as Src as proved by the experiment that treatment with Dasatinib, a protein kinase inhibitor, remarkably reduces AGR3-dependent migration. In addition, PDIA3 promotes pro-migratory phenotypes in either luminal (MCF-7) or basal breast tumor subtypes (MDA-MB-231 and HCC1937), and PDI inhibition led by 16F16 efficiently decreases initial cell spreading [94]. Similar to 16F16, another PDI inhibitor, PACMA31, could block the transendothelial migration of MDA-MB-231 cells and the contraction of collagen that affect the exposition of free thiols on integrin molecules [86]. 3,4-Methylenedioxy-*β*-nitrostyrene (MNS), whose potential target is cell surface PDI, profoundly inhibits the adhesion of TNBC cells to different ECM components via the suppression of β_1_ integrin activation and focal adhesion signaling [95].

### 5.3. Role of PDI in Breast Cancer Chemoresistance and Clinical Outcomes

In addition, the supporting role of PDIs in developing drug resistance has been introduced in cancers. For example, PDIA4 and PDIA6 are overexpressed in cisplatin-resistant lung cancer cells, suggesting that their overexpression is associated with developing resistance [68]. Additionally, it is found that PDI-associated ATF6 signaling correlates with tumor cell resistance to imatinib treatment in leukemia cells [62]. Similarly, an increased PDI expression is observed in the multidrug-resistant breast cancer cells MCF-7/AdVp3000 [96] or mitoxantrone-resistant MCF7/MX cells [97].

The expression of one of the PDI isoforms, ERp29, is reported to help increase the resistance to doxorubicin and, expectedly, decrease the doxorubicin-induced cell apoptosis in MDA-MB-231 cells [98]. The same effect is observed in MCF-7 cells, as its knockdown increases the doxorubicin cytotoxicity. The ERp29-mediated resistance to doxorubicin could be explained by the up-regulation of Hsp27. However, interestingly, similar resistance by ERp29 is not obtained in response to cisplatin and paclitaxel [98]. AGR2 is a direct target of ERα with direct binding as evidenced by chromatin immunoprecipitations. However, counterintuitively, AGR2 expression is increased in tamoxifen-treated ER-positive breast cancer rather than inhibited by tamoxifen [99]. This could be explained by the agonistic role of tamoxifen on AGR2; the resultant overexpression of AGR2 potentially contributes to tamoxifen resistance and decreased overall survival [77].

Hypoxia in the tumor microenvironment is an indicator of aggressive disease and decreased overall survival in various solid tumors. The underlying mechanism of this observation is the upregulation of genes associated with metastasis, which is, unsurprisingly, associated with poor prognosis in breast cancer. Hypoxia is a transcriptional activator of PDIs, which concurrently also upregulates the expression of human endoplasmic reticulum oxidoreduction 1-α (ERO1-α). The upregulation of ERO1-α causes significantly shorter disease-free and overall survival compared to ERO1-α negative patients. Thus, the findings strongly suggest ERO1-α as a new predictor for poor breast cancer prognosis [100]. In addition, hypoxia-inducible factor (HIF-1) is associated with poor outcomes in breast cancer [101,102]. Recently, PDI has been identified as a novel regulator of HIF-1α by directly oxidizing HIF-1α and decreasing its expression level [103]. Associated with regulating these gene signatures, PDI expression is also suggested as a predictor of poor prognosis in breast cancer. From the data obtained from Oncomine, lower PDI expression is significantly associated with a higher overall survival rate of patients with breast cancer [70]. For example, AGR2 is described as an overexpressed gene in ER-positive breast cancer, and a high level of AGR2 is associated with poor prognosis in the same type of breast cancer [77].

### 5.4. Role of PDI as Transcriptional Cofactors

The roles of PDIs have been reported as transcriptional cofactors such as ERα, nuclear factor kappa B (NF-kB), Signal Transducer and Activator of Transcription 3 (STAT3), HIF-1α, and nuclear factor (erythroid-derived 2)-like 2 (Nrf2). PDIA1 directly interacts with ERα and regulates its transcriptional activity [82,104]. Overexpression of PDI suppresses NF-kB-dependent transcriptional activity, working as a negative regulator of NF-kB [105]. PDIs such as PDIA3 (ERp57) are known to localize to the nuclear matrix and interact with STAT3 and calreticulin [106,107]. Other transcription factors are also found to be associated with PDIs, such as HIF-1α [108] and Nrf2 [109].

Interestingly, PDIA1 (P4HB) is known to affect the cell surface levels of the non-classical human leukocyte antigen (HLA-G) in breast cancer cell lines (MDA-MB-231 and MCF-7) [110]. ERO1-α, in collaboration with PDIs, suppresses antitumor immunity by regulating CXCL1, CXCL2, and MHC class I [111,112,113]. Thus, it is likely that PDIs play a role in antigen presentation in the tumor microenvironment and tumor immunorecognition.

## 6. PDI Inhibitors

### 6.1. PDI Inhibitors Categorized Depending on Binding Sites

**Figure 5:**
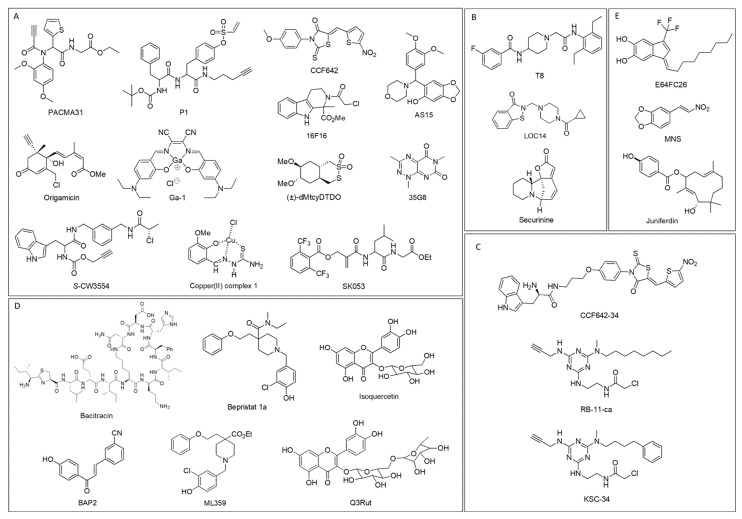
The chemical structures of small-molecule PDI inhibitors: (**A**) PDI inhibitors that likely bind to the catalytic sites of the a and a’ domains of PDI; (**B**) PDI inhibitors that bind adjacent to active sites; (**C**) selective PDIA1 inhibitors; (**D**) PDI inhibitors that likely bind to the allosteric b’ domain of PDI; and (**E**) PDI inhibitors that the binding site of PDI is not yet identified.

There is emerging evidence of a potential therapeutic role for the use of PDI inhibitors in treating various cancers. In general, PDI inhibitors work either by binding catalytic active sites selectively or non-selectively or by binding allosteric substrate binding site. Depending on the site of PDI where binding occurs, PDI inhibitors are categorized into five different groups (Figure 5). The detailed information about selectivity towards PDI isoforms, modes of action, cell-based and pre-clinical studies, and clinical trials of the PDI inhibitors are summarized in Table 1.

Most PDI inhibitors are known to or likely to bind to catalytic sites of the a or a’ domain of PDI: they include PACMA31 [114], P1 [115], 16F16 [116], AS15 [117], CCF642 [118], *S*-CW3554 [119], Origamicin [120], (±)-dMtcyDTDO [121], Ga-1 [122], 35G8 [123], Copper (II) complex 1 [124], and SK053 [125]. PACMA31 belongs to the class of propynoic acid carbamoyl methyl amides (PACMAs), and it covalently binds to the Cys400 of the active site of PDI, and it shows cytotoxicity on a broad range of human cancer cells [81]. PACMA31 has in vitro and in vivo cytotoxicity in ovarian cancer cells [114] and enhances the efficacy of sorafenib in hepatocellular carcinoma models [126]. P1 is a phenyl vinyl sulfonate compound identified as an irreversible PDI inhibitor by reacting with active site cysteines via the vinyl-sulfone electrophile [115]. P1 is 10-fold more potent than PACMA31 in inhibiting in vitro PDI activities, as demonstrated by the insulin aggregation assay. The antiproliferative effects of P1 are demonstrated in various cancer cells (UACC-257, HeLa, HepG2) in the low micromolar range. 16F16 is another small-molecule irreversible PDI inhibitor that binds to the cysteines in the active site via a chloroacetamide electrophile, and it suppresses apoptosis in cell and brain slice models of Huntington disease [116]. AS15 was recently identified as a covalent nanomolar PDI inhibitor that shows synergistic growth inhibition of glioblastoma cells when treated with the glutathione synthesis inhibitor buthionine sulfoximine (BSO) [117]. CCF642, *S*-CW3554, and Origamicin are also irreversible PDI inhibitors, showing cytotoxicity in multiple myeloma cells (CCF642 and *S*-CW3554) [118,119] and in neuroblastoma cells (Origamicin) [120].

Disulfide bond disrupting agents (DDAs) are a new class of irreversible PDI inhibitors, and dimethoxy-tcyDTDO and (±)-dMtcyDTDO are confirmed to bind to the active site cysteines of AGR2/3 and ERp44 [121]. It seems to interact with PDIA1, but its binding site on PDIA1 has not yet been determined. A Gallium (III) complex, Ga-1, is a metal-based PDI inhibitor and most likely interacts with active site His55 of PDIA3 with the cyanic group [122]. In addition to PDIA3, three other isoforms such as PDIA1, PDIA4, and PDIA6 are determined for Ga-1 to bind. Ga-1 exhibits stronger anticancer activities than cisplatin in various cancer cells (HeLa, HepG2, A549, HUVEC).

35G8, Copper (II) complex 1, and SK053 are PDI inhibitors that likely bind to active sites, but their exact binding sites are not yet determined. 35G8 inhibits proliferation in glioblastoma cells by leading to autophagy-mediated ferroptosis [123]. Copper (II) complex 1 is a metal-based PDI inhibitor and exhibits antitumor activity in various cancer cells in vitro (2008, BxPC3, PSN1, HCT-15, BCPAP, and A375) and in vivo (LLC) [124]. SK053 induces differentiation and cell death in acute myeloid leukemia cells [125].

**Table 1:** The characteristics of PDI inhibitors.

PDI Inhibitor	Specificity toward PDIs	Mode of Action	Cell-Based and Pre-Clinical Studies	Refs
PACMA31	PDIA1, PDIA2, PDIA3, PDIA4, PDIA6, TXNDC5	Binds to active site Irreversible	Exhibits cytotoxicity in ovarian cancer cells (in vitro and in vivo) Enhances efficacy of sorafenib in hepatocellular carcinoma model (in vivo) Inhibits proliferation of breast cancer cells (MCF-7, MDA-MB-231) and reduces breast cancer adhesion and migration (MDA-MB-231)	[81,86,114,126,127]
P1	PDIA1, PDIA4, PDIA6	Binds to active site Irreversible	Inhibits proliferation in low-micromolar range against six cancer cells (UACC-257, HeLa, HepG2, MCF-7, MDA-MB-231, T47D)	[115]
16F16	PDIA1, PDIA3	Binds to active site Irreversible	Suppresses apoptosis in cell and brain slice models of Huntington disease Reduces cell adhesion/migration of breast cancer cells (MCF-7, MDA-MB-231, HCC1937)	[94,116]
AS15	PDIA1	Binds to active site Irreversible	Synergistic growth inhibition of glioblastoma cells when treated with glutathione synthesis inhibitor buthionine sulfoximine (BSO)	[117]
CCF642	PDIA1, PDIA3, PDIA4	Binds to active site Irreversible	Exhibits potent effects against multiple myeloma activity (in vitro and in vivo)	[118]
*S*-CW3554	PDIA1	Binds to active site Irreversible	Reduces cell viability of multiple myeloma cell lines (MM 1.S and KMS11)	[119]
Origamicin	PDIA1	Binds to active site Irreversible	Impairs viability of neuroblastoma cells (SH-SY5Y)	[120]
(±)-dMtcyDTDO	PDIA1, AGR2, AGR3, ERp44	Binds to active site of AGR2/3 and ERp44 Irreversible	Induces cell death in breast cancer cells in vitro (MDA-MB-468) and in vivo (mice xenograft model of BT474)	[121]
Ga-1	PDIA1, PDIA3, PDIA4, PDIA6	Binds to active site of PDIA3	Enhances cytotoxicity in various cancer cells (HeLa, HepG2, MCF-7, A549 and HUVEC cells) Shows more effective tumor suppression than cisplatin in HeLa tumor-bearing mice	[122]
35G8	PDIA1	Likely binds to active site	Inhibits proliferation of glioblastoma cells Leads to autophagy-mediated ferroptosis in glioblastoma	[123]
Copper (II) complex 1	PDIA1	Likely binds to active site	Exhibits antitumor activity in various cancers in vitro (2008, BxPC3, PSN1, HCT-15, BCPAP, A375) and in vivo (LLC)	[124]
SK053	PDIA1	Likely binds to active site (a’ domain)	Induces differentiation and cell death of acute myeloid leukemia cells	[125]
T8	PDIA1	Binds adjacent to active site Reversible	Sensitizes various cancer cells to etoposide treatment (Jurkat, MDA-MB-231)	[128]
LOC14	PDIA1, PDIA3	Binds adjacent to active site Reversible	Exhibits neuroprotective effects in corticostriatal brain slice cultures Attenuates mutant huntingtin toxicity	[129,130]
Securinine	PDIA1	Binds adjacent to the active site Irreversible	Exhibits neuroprotective effects in PC12 mHTT-Q103 cells	[131]
CCF642–34	PDIA1	Selective PDIA1 inhibitor Likely binds to active site (a’ domain)	Induces apoptosis in myeloma cells but not in normal bone marrow cells Orally bioavailable and effective against multiple myeloma in vivo	[132]
RB-11-ca	PDIA1	Selective PDIA1 inhibitor Binds to active site of a domain Irreversible	Inhibits proliferation of HeLa cells in micromolar range	[133]
KSC-34	PDIA1	Selective PDIA1 inhibitor Binds to active site of a domain Irreversible	Decreases secretion of a destabilized, amyloidogenic antibody light chain at non-toxic concentrations	[134]
Bacitracin	PDIA1	Binds to b’ domain	Enhances melanoma cell death Inhibits migration and invasion of malignant glioma cells	[135,136,137,138]
BAP2	PDIA1, PDIA2	Binds to b’ domain	Reduces tumor growth in glioblastoma (in vitro and in vivo) Inhibits migration of glioblastoma cells in dose-dependent manner	[66,127]
Bepristat 1a	PDIA1	Binds to b’ domain Reversible	Blocks platelet activation in-vitro and impair platelet accumulation at vascular injury site in an in-vivo model of thrombus formation	[139]
Q3Rut	PDIA1	Binds to b’ domain Reversible	Inhibits platelet aggregation (in vitro) and thrombus formation (in vivo)	[140,141]
Isoquercetin	PDIA1	Binds to b’ domain Reversible	Inhibit platelet thrombus formation and fibrin generation in a mouse model of thrombosis Clinical trials (NCT02195232, NCT04514510)	[140,141,142]
ML359	PDIA1	Likely binds to b’ domain Reversible	Inhibits platelet aggregation No cytotoxicity in cancer cells (HeLa, HEK293, HepG2)	[143]
E64FC26	PDIA1, PDIA3, PDIA4, PDIA6, TXNDC5	Pan-style inhibitor	Exhibits anti-MM (multiple myeloma) effect (in vitro and in vivo)	[144]
MNS	Cell surface PDI	Unknown	Inhibits platelet aggregation Inhibits proliferation and reduces cell adhesion and migration in MDA-MB-231 cells	[95,145,146]
Juniferdin	PDIA1	Unknown	Inhibits replication of influenza A and B virus in cells Inhibits reduction of HIV-1 gp120	[147,148]

The typical mode of action for the PDI inhibitors in the first category, with a few exceptions, is to attack the highly susceptible cysteine residues of the active site of PDI through their electrophilic moieties such as chloroacetamide or vinyl sulfones. This covalent bond formation could disrupt the CXXC motif of the PDI domain, ultimately losing the function of PDI. The non-specific and irreversible binding characteristics of the PDI inhibitors render them lacking in selectivity towards PDI members, especially those which share the CXXC motif in the active site. For example, PACMA31 irreversibly inhibits PDIA1 but also interacts with other members such as PDIA2, PDIA3, PDIA4, PDIA6, and TXNDC5 [127]. Similarly, CCF642 is also known to bind to PDIA1, PDIA3, and PDIA4 [118].

The next group includes PDI inhibitors that bind adjacent to the active site: T8 [128], LOC14 [129], and Securinine [131]. T8 binds deeply into the two binding groves adjacent to each catalytic site by hydrophobic interaction with its diethylphenyl group [128]. T8 impairs substrate-binding without disruption of the catalytic cysteines of the active site, thus being a reversible PDI inhibitor. Likewise, LOC14 is a reversible and non-covalent PDI inhibitor. LOC14 interacts with the region adjacent to the active site, inducing the protein to adopt an oxidized conformation and inhibiting its reductase activity [129]. LOC14 shows neuroprotective effects in corticostriatal brain slice cultures [129] and attenuates mutant Huntingtin toxicity [130]. Securinine likely binds adjacent to the active site similar to T8 and LOC14, but it is an irreversible PDI inhibitor [131]. Securinine interacts with H38 and R103 of PDI, which are important to stabilize the negatively charged cysteine thiolate transition state of PDI. So, its interaction leads to the destabilization of active site C36 residue, thus inhibiting PDI irreversibly. Securinine has neuroprotective effects in a cell model of Huntington disease [131].

Different from the first two groups, which may have several PDI isoforms, including PDIA1, to target, there are specific inhibitors targeting PDIA1 only. As expected, they selectively interact with the active site of either the a domain or the a’ domain of PDIA1. CCF642-34 is an optimized analog of CCF642 and likely binds to the active site of the a’ domain of PDIA1 [132]. It improves the selectivity of CCF642 on PDIA1; thus, it is a selective PDIA1 inhibitor. CCF642-34 is an orally bioavailable and effective agent against multiple myeloma [132]. RB-11-ca and KSC-34, the optimized analog of RB-11-ca, are a-domain selective PDIA1 inhibitors, as they covalently bind to C53 of the *N*-terminal cysteine in a domain of PDIA1 [134]. RB-11-ca inhibits the proliferation of HeLa cells in the micromolar range [133]. KSC-34 demonstrates its ability to reduce the extracellular pathogenic load of the amyloidogenic antibody light-chain [134].

Instead of binding to catalytic domains of PDI, there are PDI inhibitors that bind to the allosteric b’ domain, such as Bacitracin [135], BAP2 [66,127], Bepristat 1a [139], Quercetin-3-rutinoside (Q3Rut) and Isoquercetin [140,141], and ML359 [143]. The inhibitors in this category are reversible PDI inhibitors due to their interactions with the allosteric b’ domain of PDI. Bacitracin is a peptide antibiotic and is widely used as a PDI inhibitor in research. It inhibits PDI by binding to the b’ domain. However, bacitracin is not a specific PDI inhibitor, so it exhibits many off-target effects due to lack of selectivity on PDI [135,136]. Bacitracin enhances melanoma cell death [137] and inhibits the migration and invasion of malignant glioma cells [138]. BAP2 is a PDI inhibitor containing a chalcone scaffold [66]. BAP2 reduces tumor growth in glioblastoma and inhibits the migration of glioblastoma cells in a dose-dependent manner [66,127]. Another allosteric PDI inhibitor, Bepristat 1a, blocks platelet activation and impairs platelet accumulation at vascular injury sites in a model of thrombus formation [139]. Q3Rut and isoquercetin both inhibit platelet aggregation and thrombus formation [140,141]. A flavonoid, isoquercetin, is the only PDI inhibitor that is currently undergoing clinical trials. The oral isoquercetin in phase II/III improves markers of coagulation in advanced cancer patients (NCT02195232) [142]. A similar hypothesis being tested in clinical trials in patients with sickle cell disease is that isoquercetin would diminish thrombo-inflammatory venous thromboembolism (VTE) biomarkers and attenuate the associated hypercoagulable state (NCT04514510). ML359 is a reversible PDI inhibitor that likely binds to the b’ domain, and it inhibits platelet aggregation without cytotoxicity in cancer cells (HeLa, HEK293, HepG2) [143].

The last group includes PDI inhibitors whose binding site of PDI is not yet identified. E64FC26 is pan-style PDI inhibitor affecting several PDI members, including PDIA1, PDIA3, PDIA4, PDIA6, and TXNDC5, but its binding site is not reported [144]. E64FC26 induces apoptosis and cytotoxicity in multiple myeloma cells [144]. MNS is a small-molecule synthetic PDI inhibitor that works by potentially targeting the cell surface of PDI [95,145]. MNS inhibits platelet aggregation by preventing the activation of integrin αIIbβ3 [145]. Juniferdin is a natural compound that inhibits PDIA1, although the clear mode of action is not yet determined [147]. It inhibits the replication of influenza A and B virus in cells [147] and inhibits the reduction of HIV-1 gp120 [148].

### 6.2. PDI Inhibitors in Breast Cancer

Increased numbers of PDI inhibitors have been developed for various diseases, including cancers, neurodegenerative diseases, cardiovascular diseases, and infectious diseases. Among the inhibitors, only a few PDI inhibitors have been used for breast cancer, including PACMA31, P1, 16F16, DDA, Ga-1, T8, and MNS. The findings of using PDI inhibitors in breast cancer research and the proposed mechanism of action of PDI inhibition are summarized in Table 2.

PACMA31, P1, 16F16, and DDA (±)-dMtcyDTDO are irreversible PDI inhibitors. PACMA31 is one of the popular PDI inhibitors explored for breast cancer. In particular, PACMA31 displays its inhibition effect on the cell proliferation of breast cancer cells MCF-7 and MDA-MB-231, but, more significantly, in MDA-MB-231 when the concentration exceeds 10 μM [86]. MCF-7, which lacks integrin β1 and β3 expression, has a non-invasive character, whereas MDA-MB-231, with highly expressed β1 and β3, adheres more effectively to ECM proteins as well as endothelial cells. PACMA31 significantly reduces the adhesion and migration of invasive MDA-MB-231 cells compared to 16F16 and Q3Rut [86]. It blocks the transendothelial migration of the cells by inhibiting thiol-disulfide exchanges of integrin molecules β1 and β3, thus suggesting the involvement of integrins in breast cancer progression and metastasis.

The antiproliferative activity of P1 is confirmed in breast cancer cells such as MCF-7, MDA-MB-231, and T47D with a GI_50_ of 3~4 μM [115]. It is found to have PDI isoforms (PDIA4 and PDIA6) to interact besides PDIA1, so P1 could inhibit not only endogenous PDI, but also PDIA4 and PDIA6 localized in the nucleus.

16F16 is an irreversible PDI inhibitor that impairs cell adhesion and the migration of breast cancer cells MCF-7, MDA-MB-231, and HCC1937 [94]. The spreading and attachment of breast cancer cells are strongly reduced by 16F16 treatment, and the effect of 16F16 is stronger than PACMA31. 16F16 reduces the initial rates of closure and overall scratch closure for all cell lines. Also, a reduction in pro-migratory F-actin structures, including lamellipodia, is observed in the three cell lines in treatment with 16F16 [94]. Their different phenotypic responses between PACMA31 and 16F16 in the breast cancer cells are potentially due to the difference in their primary target in PDIs. PACMA31 binds to PDIA1 more strongly than PDIA3, but 16F16 could bind to both PDIA1 and PDIA3 [116].

The anticancer efficacy of DDAs is apparent in cancers overexpressing EGFR and HER2 [121]. In particular, (±)-dMtcyDTDO shows efficient cytotoxic effects on TNBC MDA-MB-468 cells that highly express EGFR and on luminal B BT474 cells that express HER2. Their cytotoxic functions could be explained as DDAs downregulating EGFR, HER2, and HER3 and activating Death Receptors 4 and 5 (DR4/5). The oligomerization of DR4 and DR5 leads to cell death via a caspase-dependent mechanism by activating caspase 8 and 3 sequentially. This might also be due to the targeting of PDI members such as PDIA1, AGR2, and ERp44 by DDAs.

Ga-1 is a gallium (III) based compound that shows stronger anticancer activity compared to cisplatin in breast cancer cells (MCF-7) with an IC_50_ of 0.42 μM [122]. Previously, the antitumor effects of Ga (III)-based compounds were considered to be iron-dependent, disrupting cellular iron homeostasis and metabolism [149]. However, Ga-1 is proved to induce ER stress, mitochondria dysfunction, and subsequent cell death in cancer cells. Treatment with Ga-1 increases the ratio of phosphorylated eIF2α to the total eIF2α, up-regulates ATF4 and CHOP, activates the mitogen-activated protein kinases (MAPK) signaling pathway, and upregulates Bax and downregulates Bcl-2 [122]. Also, Ga-1 induced mitochondria dysfunction, including disruption of mitochondria membrane permeability, could be explained by the observation that Ga-1 increases the reactive oxygen species (ROS) level, induces morphological changes of mitochondria, and decreases mitochondria membrane potential (MMP) [122].

A reversible PDI inhibitor, T8, specifically sensitizes cancer cells to the effect of the anticancer drug etoposide at a subtoxic concentration (500 nM) [128]. Therefore, the treatment of T8 in combination with an etoposide dose dependently increases the cell death rate in TNBC MDA-MB-231 cells. The apoptotic effects of T8 in combination with etoposide could result from increased PARP cleavage and increased activity of caspase 9 and caspase 3 [128].

A synthetic PDI inhibitor, MNS, inhibits not only the proliferation of human breast cancer MDA-MB-231 cells (IC_50_ of 14 μM) but also the adhesion, migration, and invasion of the cells [95,146]. MNS works potentially by blocking cell surface PDI to inhibit β_1_ integrin activation, thus affecting cell adhesion and migration. Additionally, the formation of focal adhesion complex and actin stress fiber networks are disrupted in the treatment of MNS as MNS inhibits the phosphorylation of focal adhesion kinase (FAK) and paxillin [95,146].

## 7. Concluding Remarks and Future Perspectives

PDIs play a critical role in ER proteostasis by assisting protein folding as an essential catalyst for disulfide bonds and as a chaperone. PDI members are upregulated alongside other UPR proteins in cancers, highlighting the importance of PDI in regulating cancer cell survival. Due to the protective role of PDIs in cancer cells, the prolonged inhibition of PDIs has emerged as a promising approach to induce excessive ER stress in cancer cells, leading to apoptosis in cancers.

PDI proteins are mainly localized in the endoplasmic reticulum, but are also found on cell surfaces, in the nucleus, in the extracellular space, or in mitochondria. Although the exact functions of PDIs at different subcellular localizations are not yet fully demonstrated, it seems to be apparent that PDIs at different localizations have different functions in cancers. PDIs localized in the endoplasmic reticulum are involved in UPR pathways that determine cell survival or cell death through redox regulation of UPR stress receptors such as IRE1 [57], PERK [57], or ATF6 [62], and regulation of ERAD and autophagy. PDIs located at the cell surface are involved in cell adhesion and migration as evidenced in breast cancer [78,93,95] or glioblastoma [138,150]. Inhibition of extracellular PDIs could impair cell adhesion and migration by inhibiting the activation of metalloproteases and integrins. PDIs at mitochondria are suggested to regulate endoplasmic reticulum and mitochondrial calcium dynamics [151].

In this review, several PDI members, including PDIA1 (P4HB), might be suggested as interesting molecular targets for TNBC as evidenced by the overexpression in the TNBC subtype compared to other breast cancer subtypes and the superior inhibition effects on the adhesion and migration of TNBC cells than other subtypes. Their functions as transcription cofactors such as ERα, NF-kB, or Nrf2 are interesting because they seem to be involved in antigen presentation in the tumor microenvironment and tumor immunorecognition.

Many PDI inhibitors are irreversible inhibitors that likely bind to cysteine residues in catalytic domains or affect the cysteine residues in the active site of PDI. Due to this binding characteristic, these PDI inhibitors are able to interact with other PDI isoforms or proteins that share cysteine residues in the active site, leading to a non-selective character. The non-selective PDI inhibitors might result in off-target effects. Recently, more reversible PDI inhibitors or PDIA1-specific inhibitors have been developed. These inhibitors might not have selectivity issues related to off-target effects. However, further research is needed to clearly elucidate the effects of irreversible, non-selective PDI inhibitors and reversible PDI inhibitors in cancers in terms of off-target activities, potency, toxicity, etc.

There were few studies exploring PDIs in breast cancers previously, but now an increased number of reports suggest the involvement of PDIs in tumorigenesis, metastasis, drug resistance, and poor prognosis in breast cancer, including TNBC. However, there is no clear evidence of which specific PDIs are impacting each process nor of their exact mechanisms of action. In order to develop PDI inhibitors targeting specific breast cancer subtypes such as TNBC, continuous efforts are required to identify the exact roles and mechanisms of specific PDI isoforms involved in tumorigenesis, metastasis, drug resistance, and antigen presentation in TNBC.

## Figures and Tables

**Table 2:** The PDI Inhibitors Used in Breast Cancer Research.

PDI Inhibitor	Findings in Breast Cancer Research (Cell-Based and Pre-Clinical Studies)	Mechanism of Action of PDI Inhibitors (Proposed Pathways)
PACMA31	Inhibits proliferation of breast cancer cells (MCF-7, MDA-MB-231). Highly metastatic MDA-MB-231 breast cancer cell lines adhere to endothelial cells are more effective than non-invasive MCF-10A and MCF-7 cell lines. The attachment of MDA-MB-231 to the endothelium can be attenuated by PDI inhibitors.	The transendothelial migration of MDA-MB-231 cells can be blocked by PDI inhibitors that inhibit thiol-disulfide exchange of integrin molecules β_1_ and β_3_.
P1	Inhibits cell proliferation of breast cancer cells (MCF-7, MDA-MB-231, T47D) in low micromolar range of GI_50._	Affects cell viability by inhibiting endogenous PDIA1, PDIA4, and PDIA6 localized in nucleus.
16F16	Reduces cell adhesion/migration of breast cancer cells (MCF-7, MDA-MB-231, HCC1937). The spreading and attachment of breast cancer cells are strongly reduced by 16F16 treatment, and the effect of 16F16 is stronger than PACMA31. 16F16 reduces initial rates of closure and overall scratch closure for all cell lines.	Impairs cell adhesion and migration of breast cancer cells by affecting pro-migratory F-actin structures including lamellipodia.
DDAs	The anticancer efficacy of DDAs is apparent in cancers overexpressing EGFR and HER2. Induces cell death in breast cancer cells in vitro (MDA-MB-468) and in vivo (mice xenograft model of BT474).	DDAs triggers DR4 and DR5-mediated activation of caspase 8 and 3 to cause apoptosis.
Ga-1	Inhibits cell proliferation of MCF-7 with nanomolar range of IC_50._	Ga-1 induces ER stress, mitochondria dysfunction and subsequent cell death. Ga-1 induced apoptosis is evidenced by upregulation of ATF4 and CHOP, subsequent activation of MAPK signaling pathway, and up-regulation of Bax as well as down-regulation of Bcl-2. Ga-1 induced mitochondria dysfunction is evidenced by increased ROS level, induced morphological changes of mitochondria, and decreased MMP.
T8	Combination with etoposide dose-dependently increases cell death rate in MDA-MB-231 cells.	Apoptosis is evidenced by increased PARP cleavage, caspase 9 and caspase 3 activity.
MNS	Inhibits proliferation in MDA-MB-231 cells with IC_50_ of 14 μM. Inhibits adhesion of TNBC cell lines (MDA-MB-231) to different ECM components.	MNS works potentially by blocking cell surface PDI to inhibit β_1_ integrin activation, affecting on cell adhesion and migration. Also, MNS inhibits phosphorylation of FAK and paxillin to disrupt the formation of focal adhesion complex and actin stress fiber networks.
